# Recent Applications of DNA Microarray Technology to Toxicology and Ecotoxicology

**DOI:** 10.1289/ehp.8194

**Published:** 2005-08-09

**Authors:** Teresa Lettieri

**Affiliations:** Laboratory of Molecular Ecotoxicology, Inland and Marine Water Unit, Institute for Environment and Sustainability, Joint Research Centre of the European Commission, Ispra, Italy

**Keywords:** ecotoxicology, gene expression profile, genetic signature, microarray, toxicogenomics, toxicology

## Abstract

Gene expression is a unique way of characterizing how cells and organisms adapt to changes in the external environment. The measurements of gene expression levels upon exposure to a chemical can be used both to provide information about the mechanism of action of the toxicant and to form a sort of “genetic signature” for the identification of toxic products. The development of high-quality, commercially available gene arrays has allowed this technology to become a standard tool in molecular toxicology. Several national and international initiatives have provided the proof-of-principle tests for the application of gene expression for the study of the toxicity of new and existing chemical compounds. In the last few years the field has progressed from evaluating the potential of the technology to illustrating the practical use of gene expression profiling in toxicology. The application of gene expression profiling to ecotoxicology is at an earlier stage, mainly because of the the many variables involved in analyzing the status of natural populations. Nevertheless, significant studies have been carried out on the response to environmental stressors both in model and in nonmodel organisms. It can be easily predicted that the development of stressor-specific signatures in gene expression profiling in ecotoxicology will have a major impact on the ecotoxicology field in the near future. International collaborations could play an important role in accelerating the application of genomic approaches in ecotoxicology.

Gene expression is a sensitive indicator of toxicant exposure, disease state, and cellular metabolism and thus represents a unique way of characterizing how cells and organisms adapt to changes in the external environment. The measurement of gene expression levels upon exposure to a chemical can both provide information about the mechanism of action of toxicants and form a sort of “genetic signature” from the pattern of gene expression changes it elicits both *in vitro* (Burczynski et al. 2000; Waring et al. 2001) and *in vivo* (Hamadeh et al. 2002). The development of such gene expression signatures would allow fast screening of unknown or suspected toxicants on the basis of their similarity to known toxicants.

The possibility of analyzing the effect of chemicals and environmental stressors on a large number of genes in a single experiment has led to the development of the field of toxicogenomics. Proponents of toxicogenomics aim to apply both mRNA and protein expression technology to study chemical effects in biological systems (Afshari et al. 1999; Lovett 2000; Olden and Guthrie 2001).

The availability of the complete human genome and of the genome of several other organisms (NCBI 2005b) allows the application of microarray technology to several model organisms (from bacteria, to yeast, to fish) and mammalian cell lines.

In this review I evaluate the potential of microarray technology for ecotoxicology. I briefly review recent applications of DNA microarray to toxicology and analyze how the field of ecotoxicology could benefit from the experience already gained from toxicology.

I describe examples of the contribution of the technique in addressing important ecotoxicology problems as well as problems and limitations associated with the technique. Finally, I suggest future paths for more extensive application of microarray to ecotoxicology.

This is not a comprehensive review of the current state of the art in DNA microarray technology; several exhaustive reviews are available on both the practical aspects of DNA microarrays and the analysis of data (Knudsen 2004; Schena 1999, 2003; Schulze and Downward 2001).

## Overview of Gene Expression Analyses

The field of DNA microarray has evolved from Ed Southern’s key insight (Southern 1975) 25 years ago showing that labeled nucleic acid molecules could be used to interrogate nucleic acid molecules attached to a solid support. The resulting Southern blot is considered to be the first DNA array (Southern 2000). It was only a small step to improve the technique to filter-based screening of clone libraries, which introduced a one-to-one correspondence between clone and hybridization signal (Grunstein and Hogness 1975). The next advance was the use of gridded libraries stored in microtiter plates and stamped onto filters in fixed positions. With this system, each clone could be uniquely identified and information about it accumulated. Several groups explored expression analysis by hybridizing mRNA to cDNA libraries gridded on nylon filters. The subsequent explosion of array technologies was sparked by two key innovations. The first was the use of nonporous solid support, such as glass, which has facilitated the miniaturization of the array and the development of fluorescence-hybridization detection (Lockhart et al. 1996; Schena et al. 1995, 1996). The second critical innovation was the development of methods for high-density spatial synthesis of oligonucleotides, which allows the analysis of thousands of genes at the same time. Recently, a significant technical achievement was obtained by producing arrays with more than 250,000 oligonucleotides probes or 10,000 different cDNAs per square centimeter (Lipshutz et al. 1999). DNA microarrays are fabricated by high-speed robots, generally onto glass. Because the DNA cannot bind directly to the glass, the surface is first treated with silane to covalently attach reactive amine, aldehyde, or epoxies groups that allow stable attachment of DNA, proteins, and other molecules.

**Figure 1:**
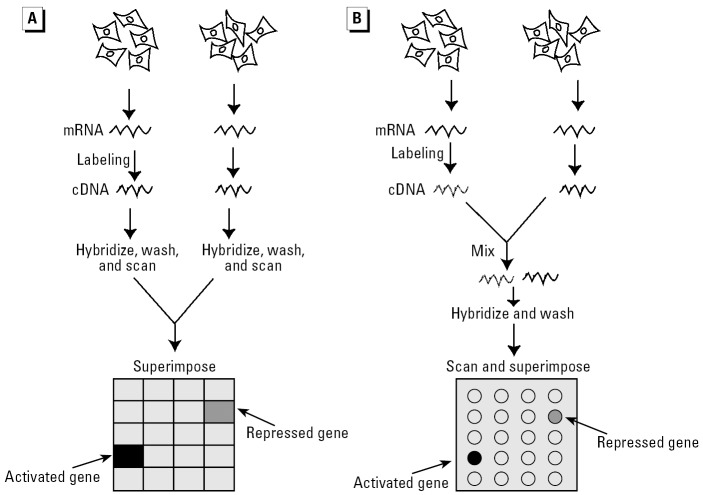
Gene expression analyses by microarray. (*A*) One-color expression analysis uses a single fluorescent label and two arrays to generate expression profiles for two cell or tissue samples (test and reference samples). Activated and repressed genes are obtained by superimposing images obtained by the two arrays. (*B*) Two-color expression analysis uses two different fluorescent labels and a single array to generate expression profiles for the test and reference samples. Activated and repressed genes are obtained by superimposing images generated in different channels on a single array. In both cases the monochrome images from the scanner are imported into software in which the images are pseudocolored and merged. Data are viewed as a normalized ratio in which significant deviation from 1 (no change) indicates increased (> 1) or decreased (< 1) level of gene expression relative to the reference sample.

The nucleic acid microarrays use short oligonucleotides [15–25 nucleotides (nt)], long oligonucleotides (50–120 nt), and PCR-amplified cDNAs (100–3,000 bp) as array elements. The short oligonucleotides are used primarily for the detection of single-nucleotide polymorphisms (SNPs). Because this application requires the discrimination of only one mismatch, the presence of a short oligonucleotide maximizes the destabilization caused by mis-pairing (Lockhart et al. 1996). Conversely, the PCR-amplified cDNAs produce strong signals and high specificity (DeRisi et al. 1996). The cDNA elements are readily obtained from cDNA libraries and are typically used for organisms for which only a limited part of the whole genome information is available. The long nucleotides offer strong hybridization signal, good specificity, unambiguous sample identification, and affordability (Hughes et al. 2000; Kane et al. 2000; Schena et al. 1998). All these advancements have allowed gene arrays to become a standard tool in molecular toxicology. With this technology, cells or tissues are exposed to toxicants, and then gene expression is measured by collecting mRNA, converting mRNA to labeled cDNA, hybridizing it to the DNA array, staining it with an appropriate dye, and visualizing the hybridized genes using a fluorometer (DeRisi et al. 1996; Lashkari et al. 1997; Schena et al. 1995) (Figure 1). The raw data are analyzed using bioinformatics software and databases. The aim is to obtain meaningful biological information such as patterns of relative induction/repression levels of gene expression, participation in biochemical pathways, and (in the most favorable cases) “genetic signatures.”

## Recent Applications of DNA Microarrays to Toxicology

The field of toxicogenomics has progressed rapidly since the application of DNA chips to toxicology was proposed in the late 1990s (Afshari et al. 1999). Publications have evolved from evaluating the potential of the technology (Burchiel et al. 2001; Fielden and Zacharewski 2001; Nuwaysir et al. 1999; Simmons and Portier 2002; Smith 2001; Tennant 2002; Ulrich and Friend 2002) to illustrating the practical use of gene expression profiling in toxicology (Bartosiewicz et al. 2001; Bulera et al. 2001; Hamadeh et al. 2002; Waring et al. 2001).

Waring et al. (2001) analyzed the hepatic effects of a new chemical substance that inhibits the expression of cellular adhesion proteins. They treated rats for 3 days and then performed the microarray analyses on RNA from livers of treated animals. The comparison of the gene expression profile with a database of profiles of known hepatotoxins indicated that hepatic toxicity of the new chemical is mediated by the aryl hydrocarbon nuclear receptor. Hamadeh et al. (2002) analyzed the patterns of gene expression in liver tissue taken from rats exposed to different chemicals. Their analysis revealed similarities in gene expression profiles between animals treated with different chemicals belonging to the same class of compounds (peroxisome proliferators). In contrast, animals treated with a different class of compounds (enzyme inducers) showed a very distinctive gene expression profile.

To expand the use of microarray technology in toxicology, several national and international initiatives have been developed to better standardize and harmonize the technology. One of the early concerns about the use of DNA microarray in toxicology has been how to properly compare experiments that use a wide variety of commercial and proprietary platforms, protocols, and analysis methods. In the United States, the National Institute of Environmental Health Sciences (NIEHS) has created the National Center for Toxicogenomics (NCT) to provide a reference system of genomewide gene expression data and to develop a knowledge base of chemical effects in biological systems (Tennant 2002). The NCT has conducted some proof-of-principle experiments to establish signature profiles of known toxicants and to link the pattern of altered gene expression to specific parameters of conventional indices of toxicity (Hamadeh et al. 2002). These studies show that it is possible to identify a signature of expressed gene patterns after exposure to a given toxicant (Tennant 2002).

The Health and Environmental Sciences Institute (HESI) of the International Life Sciences Institute (ILSI) has coordinated an international study involving more than 30 pharmaceutical companies and governmental and academic institutions to evaluate the harmonization of gene expression data and analyses (Pennie et al. 2004). In the ILSI Application of Genomics to Mechanism-Based Risk Assessment project, common pools of RNA were analyzed in more than 30 different laboratories using both similar and different technical platforms. Overviews of the design and objectives of the experimental program and more technical articles have been published in the mini-monograph *Application of Genomics to Mechanism-Based Risk Assessment* (Environmental Health Perspectives 2004). Amin et al. (2004) identified gene markers of renal toxicity, and Thompson et al. (2004), markers of cisplatin nephrotoxicity. Two research groups performed an interlaboratory evaluation of clofibrate-induced gene expression changes in rat liver (Baker et al. 2004) and of rat hepatic gene expression changes induced by methapyrilene (Waring et al. 2004). In addition three research groups have published overviews on the interlaboratory collaborations to evaluate the effects of nephrotoxicants (Kramer et al. 2004), genotoxic chemicals (Newton et al. 2004), and hepatotoxicants (Ulrich et al. 2004) on gene expression.

The experimental programs have shown that *a*) patterns of gene expression relating to biological pathways are robust enough to allow insight into mechanisms of toxicity, *b*) gene expression data can provide meaningful information on the physical location of the toxicity, *c*) dose-dependent changes can be observed, and *d*) concerns about oversensitivity of the technology may be unfounded (Pennie et al. 2004).

Very recently, DNA microarrays have been used to develop a much deeper insight into the mechanism of chemical toxicity at the molecular level. Andrew et al. (2003) used cDNA microarrays to compare the effects of arsenic, nickel, chromium, and cadmium on the expression of 1,200 human genes in human bronchial BEAS-2B cells. Cells were exposed both to low doses of the different metals and to a cytotoxic dose of sodium arsenite. Metal exposure modified only a small subset of the 1,200 genes, and each metal modified the expression of a largely unique set of genes; thus, these results could provide the basis for the development of metal-specific biomarkers. Exposure to low concentrations of sodium arsenite modified the expression of genes involved in transcription factors, inflammatory cytokines, kinases, and DNA repair. High doses of sodium arsenite gave a very different profile, modifying the expression levels of genes codifying for heat-shock proteins and other genes involved in stress-response pathways. The researchers suggested that this change in gene expression profiles represents a switch from a survival-based biological response at the lower dose to a cell-death–inducing apoptotic response at the higher dose.

Gene expression profiling has been used to show that the specific genes repressed or induced upon exposure to a toxic stress vary depending on the cell type and the type of toxicants to which the cells were exposed (Troester et al. 2004). The researchers cultured separate breast cancer cell lines known to have distinct responses to two chemotherapeutic drugs: doxorubicin (DOX), and 5-fluorouracil (5-FU). Different cell lines (two basal-like and two luminal epithelial) were treated with toxic concentrations of DOX and 5-FU, and then mRNA was extracted and analyzed. Gene expression profiling identified those genes that had been up- or down-regulated and showed a characteristic pattern of gene expression in response to DOX and 5-FU in each cell type. Detailed analyses identified a subset of 100 genes that could be used to differentiate between DOX-treated and 5-FU–treated samples.

Ezendam et al. (2004) fed Brown Norway rats with two different concentrations (low and high doses) of hexachlorobenzene (HCB) for 4 weeks, and then mRNA from several tissues was collected and analyzed. The most significant changes in gene expression, relative to the control group, occurred in spleen, followed by liver, kidney, and mesenteric lymph nodes. The gene expression profiling confirmed known effects of HCB on the immune system and induction of enzymes involved in drug metabolism and reproduction. In addition they found new up-regulated genes encoding proinflammatory cytokines, antioxidants, acute-phase proteins, complements, chemokines, and cell adhesion molecules.

A recent article clearly highlights one of the problems with using DNA microarrays. To analyze the effect of sampling differences on transcriptional profiling, investigators treated male Fischer 344 rats with toxic and nontoxic doses of acetaminophen and took liver samples of their left and median lobes (Irwin et al. 2005). Transcript profiling using microarrays showed clear differences between the left and median lobes of liver, both at low doses and at doses that cause hepatotoxicity. The two lobes of liver showed clear differences both in the pattern of gene expression and in the total number of repressed or enhanced genes.

## Public Databases for DNA Microarray Experiments

Because of the various methodologies for arraying genes and assessing mRNA expression levels, and different bioinformatics tools for the management and analyses of the data, investigators quickly realized the need to establish standards for recording and reporting microarray-based gene expression data. To this end, the Minimum Information about a Microarray Experiment (MIAME) guidelines (Brazma et al. 2001) have been developed at the European Bioinformatics Institute (EBI). This standard describes the minimum information required to ensure that microarray data can be easily interpreted and that results derived from its analysis can be independently verified.

Several public repositories of microarray gene expression data have been developed to store the results of array experiments: Array-Express (Brazma et al. 2003) in Europe, Gene Expression Omnibus (GEO) in the United States (Edgar et al. 2002), and the Center for Information Biology Gene Expression Database (CIBEX) (Ikeo et al. 2003) in Japan. Several journals already require an accession number (indicating that a data set has been submitted to one of these public databases) before publication, and there are increasing calls for mandatory submission of microarray data to a public database before publication (Ball et al. 2004).

Several initiatives aim to extend the scope of public databases of microarray data to incorporate toxicology and biologic end points. These toxicogenomics databases are being developed with the goal of creating a knowledge base to support genomic applications in hazard identification (Mattes et al. 2004). Two international consortia are developing public toxicogenomics databases with extensive cross-links to existing biological information and annotation: Tox-MIAMExpress is being developed at EBI, and the Chemical Effects in Biological Systems (CEBS) database (Waters et al. 2003) is being developed at NCT (Table 1). The CEBS knowledge base is designed to meet the information needs of “systems toxicology” involving the study of perturbation by chemicals and stressors, monitoring changes in molecular expression and conventional toxicologic parameters, and iteratively integrating biological response data to describe the functioning organism. If successfully implemented with the appropriate depth of data content, such databases could serve as robust resources for advanced queries.

Publicly available software tools have been developed to help in the interpretation and analyses of DNA microarray data. ArrayTrack (Tong et al. 2004), developed at the National Center for Toxicological Research (NCTR) of the Food and Drug Administration, is public microarray data management and analysis software. It provides data management, visualization tools, and functional information about genes, proteins, and pathways drawn from various public biological databases for facilitating data interpretation.

## Recent Applications of DNA Microarrays to Ecotoxicology

One challenge facing ecotoxicology is to understand the mechanism of action of toxicants on living organisms (Snape et al. 2004). Such knowledge would help to develop predictive simulation models of toxic effects, to link molecular biomarkers with population-level effects, and then to anticipate ecologic risk assessment issues for new chemicals. Gene expression profiles represent the primary level of integration between environmental factors and the genome, providing the basis for protein synthesis, which ultimately guides the response of organisms to external changes. Thus, the analysis of gene expression changes is a powerful tool both to diagnose major stressors in natural populations and to analyze the mechanisms of such stress responses.

Using gene expression profiles in ecotoxicology requires careful planning of experimental protocols that should take into proper account possible sources of variations in gene expression in natural populations. In fact, differences in gene expression due to sex, genotype, age, and intrinsic genetic variability can be quite substantial (Jin et al. 2001; Oleksiak et al. 2002; Ranz et al. 2003; Townsend et al. 2003).

DNA microarray technology has been applied extensively to the analyses of natural and anthropogenic factors in yeast for which whole-genome chips have been available for a few years (Causton et al. 2001; Gasch et al. 2000; Momose and Iwahashi 2001). Causton et al. (2001) analyzed how the whole genome of yeast is remodeled in response to environmental stressors such as temperature, pH, oxidation, and nutrients. The stress response was dependent on the level of the stress and showed an additive effect for multiple stressors. Similar results were found when using different stressors such as temperature shock, amino acid starvation, nitrogen source depletion (Gasch et al. 2000), and cadmium (Momose and Iwahashi 2001). The same approach has been used to characterize the alteration of gene expression in yeast induced by the pesticide thiuram (Kitagawa et al. 2002). The results obtained for stress response in yeast likely will provide a reference frame for similar experiments with more complex organisms.

Custom-made microarrays have been used to understand responses to endocrine modulators in zebrafish (Hoyt et al. 2003). Zebrafish embryos were exposed *in vitro* to the environmental contaminant 4-nonylphenol (4NP). The gene expression profiling (using a custom-made microarray with 230 genes) identified a set of 9 genes associated with the function of estrogen response that is indicative of embryo exposure to 4NP even at low concentrations. A similar approach has been used to study the gene expression profiling in response to environmental stressors in the typical plant model organism *Arabidopsis thaliana*. Using a cDNA microarray containing about 7,000 genes, Seki et al. (2002) determined the expression profiles under drought, cold, and high-salinity conditions. Their analysis revealed a subset of 53, 277, and 194 genes that were differentially expressed > 5-fold after cold, drought, and high-salinity treatments, respectively. A set of 22 stress-inducible genes was found to respond to all three stress conditions. In a similar study the oxidative stress caused by high ozone concentrations has also been analyzed in *A. thaliana* (Ludwikow et al. 2004) with DNA microarray. A review of the applications of DNA microarrays for expression profiling under stress conditions in *A. thaliana* has been recently published by Seki et al. (2004) of the Riken Genomic Sciences Center in Kanagawa, Japan.

The application of gene expression profiles is not limited to model organisms for which the complete (or almost complete genome) is available. Several strategies are available to apply a genomic approach to species for which only a limited amount of genomic information is available. One possibility is heterologous hybridization. In fact, because of the length of the probes, cDNA microarrays can be used in heterologous hybridizations across strains and closely related species as long as sequence divergence is limited for a given gene (Rise et al. 2004b). For example, Hittel and Storey (2001) have used this approach to study the molecular basis of traits, such as hibernation, not present in model species. More recently, Renn et al. (2004) have used heterologous hybridization to study gene expression profiling across a wide range of different species of African cichlid fish.

Another possible approach consists of identifying stress-induced genes using special techniques based on PCR, such as differential display PCR, suppressive subtractive hybridization PCR, and representational difference analyses. The application of these techniques to ecotoxicology has been reviewed recently by Snell et al. (2003).

Gracey et al. (2001) used cDNA microarrays to identify hypoxia-induced genes in a nonmodel fish for which sequence data were unavailable. Their analysis revealed that although some changes in gene expression mirror the changes that occur in mammals, novel genes are differentially expressed in fish and tissue-specific patterns of gene expression occur during hypoxia.

Larkin et al. (2003) described an expression profiling model system for endocrine-disrupting compounds that mimic estrogens. The research group created a gene array by cloning 30 genes from sheepshead minnows. The genes had been previously identified by differential display reverse transcriptase PCR, a method that screens thousands of RNA messages to identify genes that are turned on or off by specific treatments. They treated the fish with a constant concentration of weak and strong environmental estrogens and then determined which genes were differentially expressed in the livers of treated and control fish. They found a group of genes that were up-regulated by all the tested compounds, while other genes showed differential expression only in response to a specific compound. Exposure to different concentrations of the strong estrogen 17α-ethynyl estradiol revealed that changes in gene expression levels are dose sensitive and that exposure thresholds vary for different genes. A similar approach has been used to identify alterations in gene expression due to exposure to androgen hormones in largemouth bass fishes (Blum et al. 2004).

Williams et al. (2003) used a cDNA microarray-based approach to analyze the expression level changes of recognized biomarkers in a relevant fish species, European flounder (*Platichthys flesus*). They arrayed 160 genes, of which 110 were already documented in the literature as biomarkers of toxic response in fishes and mammals. Five adult males and five adult feral females *P. flesus* were caught from the Tyne (polluted) and the Alde (unpolluted) estuaries in the United Kingdom. Gene expression analysis revealed that 11genes were expressed differently in males between the Tyne and Alde. Such differences were not statistically significant in females, probably because of interindividual variations. Vitellogenin levels differed radically among the female fish, suggesting that their reproductive cycles were at different stages.

Despite the lack of extensive genomic data, invertebrates are the subject of increased interest. Because of their characteristics, estuarine amphipods typically are used to assess the ecologic risk associated with contaminated sediments. Perkins and Lotufo (2003) isolated several genes from *Leptocheirus plumulosus* and developed a quantitative assay to measure the effects of water exposure to 2,4,6-trinitrotoluene and phenanthrene on gene expression. They found that expression of the genes for actin and a retrotransposone element, hopper, were dependent on the exposure and tissue concentrations of those chemicals. Diener et al. (2004) have optimized a protocol for differential display PCR to investigate gene expression in *Daphnia magna*. Their protocol requires submicrogram amounts of total RNA and fewer than 10 animals and thus could provide a significant technical improvement for gene expression analyses of toxicant exposure.

Several efforts are focusing on the detection of pathogen infection in different animal species. Panicker et al. (2004) developed a gene array for detection of pathogenic *Vibrio* species, which are natural inhabitants of warm coastal waters and shellfish. Recently, microarray analysis has also been applied successfully to identify molecular markers of pathogen infection in salmon (Rise et al. 2004a). This analysis identified transcripts induced and repressed by the pathogen, thus providing insights into the host response to the infection and into the mechanisms used by the pathogen to evade such response.

## Limitations of DNA Microarrays in Ecotoxicology

The enormous potential that lies in the successful incorporation of genomic data into ecotoxicology faces several challenges. The major challenge is the difficult task of taking into account intrinsic sources of variability in gene expression levels due to different physiologic states, age, sex, and genetic polymorphisms in natural populations. Somewhat related is the additional problem of properly interpreting array data in the presence of such large intrinsic variations and then relating changes in gene expression to significant ecotoxicologic parameters (i.e., at the population level) such as survival, growth, and reproduction. A second major limitation is the high cost associated with the technology itself. These costs render repeat measures very expensive, and thus often only limited experimental data are available.

The expression of certain genes can vary considerably even under tightly controlled experimental conditions. Fay et al. (2004) observed that around 400 genes were differentially expressed when analyzing nine different strains of *Saccharomyces cerevisiae*. To minimize the effect of genetic polymorphisms on gene expression levels, investigators usually detemine the toxic properties of chemicals using inbred strains of mice and rats or well-characterized strains of yeast. In natural populations of non-model organisms, two approaches can be used to determine the “normal” gene expression patterns (Neumann and Galvez 2002). Pooling RNA samples from a large number of individuals in the control group will provide a measure of the mean gene expression response. This approach has the advantage of requiring a low number of microarrays, thereby reducing the overall cost of the experiment, but it does not provide any information about the distribution of responses in the natural population. The other, more expensive approach consists of measuring gene expression patterns for each individual in the control population. This approach makes it possible to obtain both the mean expression pattern and its distribution.

An additional problem is the limited availability of DNA arrays for nonmodel organisms (Table 2). Even if several techniques are available to identify subsets of genes that respond to environmental stressors, the lack of whole-genome arrays does not allow use of the full potential of microarrays. From this point of view, it is reassuring that the number of fully sequenced genomes is expanding very fast. For example, the recent sequencing of the diatom algae *Thalassiosira pseudonana* (Armbrust et al. 2004) has added to the list a very important organism for ecotoxicology studies.

One of the best ways to advance the field is for investigators to focus on more precise objectives (Snell et al. 2003): identify conserved genes that are up-regulated in response to toxicant exposure, determine how these gene expression profiles can be used to diagnose stressors, and identify those genes that are most informative to incorporate into stress gene arrays.

## Conclusions

The application of gene expression analysis to toxicology is now a mature science. The field has rapidly progressed from the proof-of-principle phase to actual applications, and gene expression profiling is now being used in screening for toxicity of new and existing chemical compounds. It can be predicted with confidence that in the future, gene expression data will also be incorporated in the regulatory arena as soon as the relevant agencies establish the regulatory framework. The national and international collaborations (e.g., HESI and NCT) that have tested the capabilities and interlaboratory reproducibility of gene expression data have played an important role in this rapid progress.

The application of this technology to ecotoxicology is at an earlier stage compared with that of toxicology, mainly because of the more complex problem and the many variables involved in analyzing the status of natural populations in a real ecosystem. Investigators have obtained good results using DNA microarrays in ecotoxicology both with model and with nonmodel organisms. In particular, stressor-specific microarrays have now been developed, and more will likely be available in the near future.

International collaborations will play an important role in accelerating the pace of discoveries and the application of gene chip technology to urgent problems in ecotoxicology. The experience gained from the ILSI genomic project (Pennie et al. 2004) clearly shows the advantages of interlaboratory comparison tests in terms of validation of the technology. Such international collaborations will help to spread best laboratory practices and expertise and should speed up the adoption of these new techniques by research laboratories and by the regulatory agencies.

Despite all the complications described in this article, development of stressor-specific signatures in gene expression profiling in ecotoxicology will have a major impact on the field.

## Figures and Tables

**Table 1:** List of cited databases and repository services.

Acronym	Full name	Website and reference
ArrayExpress	ArrayExpress at EBI	www.ebi.ac.uk/arrayexpress (EBI 2005a)
GEO	Gene Expression Omnibus	www.ncbi.nlm.nih.gov/geo (NCBI 2005a)
CIBEX	Center for Information Biology Gene Expression Database	cibex.nig.ac.jp (National Institute of Genetics 2005)
Tox-MIAMExpress	Toxicogenomics MIAMExpress	www.ebi.ac.uk/tox-miamexpress (EBI 2005b)
CEBS	Chemical Effects in Biological Systems	cebs.niehs.nih.gov (NIEHS 2005)
ArrayTrack	NCTR’s Center for Toxicoinformatics-ArrayTrack	www.fda.gov/nctr/science/centers/toxicoinformatics/ArrayTrack (NCTR 2005)

**Table 2:** List of commercially available gene chips.

Organism	Company	Organism	Company
*Escherichia coli*	Affymetrix	*Bos taurus*	Affymetrix
	Sigma-Genosys	*Canis familiaris*	Affymetrix
	Takara	*Mus musculus*	Affymetrix
*Bacillus subtilis*	Affymetrix		Agilent
	Sigma-Genosys		Sigma-Genosys^b^
*Pseudomonas aeruginosa*	Affymetrix		SuperArray^b^
*Helicobacter pylori*	Sigma-Genosys	*Rattus norvegicus*	Affymetrix
*Mycobacterium tuberculosis*	Sigma-Genosys		Agilent
*Staphylococcus aureus*	Affymetrix		SuperArray
*Synechocystis* sp.	Takara		Takara^c^
*Saccharomyces cerevisiae*	Affymetrix Agilent	*Homo sapiens*	Affymetrix Agilent
*Magnaporthe grisea*	Agilent		Genotypic
*Plasmodium falciparum*	Affymetrix		Sigma-Genosys^b^
*Anopheles gambiae*	Affymetrix		SuperArray^b^
*Caenorhabditis elegans*	Affymetrix	*Arabidopsis thaliana*	Affymetrix
*Drosophila melanogaster*	Affymetrix		Agilent
*Micropterus salmoides*	EcoArray^a^		Takara
*Pimephales promelas*	EcoArray^a^	*Glycine max* L.	Affymetrix
*Danio rerio*	Affymetrix	*Oryza sativa*	Agilent
	Agilent	*Hordeum vulgare* L.	Affymetrix
*Xenopus laevis*	Affymetrix	*Vitis vinifera*	Affymetrix

Company addresses are as follows: Affymetrix: Santa Clara, California, USA; Agilent: Palo Alto, California, USA; EcoArray: Alachua, Florida, USA; Genotypic: Bangalore, India; Sigma-Genosys: The Woodlands, Texas, USA; SuperArray: Frederick, Maryland, USA; Takara: Otsu Shiga, Japan.

a The bass and fathead minnow chips contain many genes important for toxicology response, including vitellogenin and several cytochrome P450s, among others.

b The cDNA or oligo microarrays have been designed to profile the expression of multiple genes involved in a specific biological pathway, or genes with similar functions or structural features. Mouse and human cDNA microarrays are also available for toxicology and pharmacology applications. This type of array is designed to determine the expression profile of genes responsible for metabolism of endogenous and exogenous compounds.

c This is a glass slide array immobilized with approximately 390 cDNA fragments of rat genes related to the stress and toxicity responses.
